# No substantial neurocognitive impact of COVID-19 across ages and disease severity: a multicenter biomarker study of SARS-CoV-2 positive and negative adult and pediatric patients with acute respiratory tract infections

**DOI:** 10.1007/s15010-024-02406-7

**Published:** 2024-10-01

**Authors:** Johannes Ehler, Felix Klawitter, Friedrich von Möllendorff, Maike Zacharias, Dagmar-Christiane Fischer, Lena Danckert, Rika Bajorat, Johanna Hackenberg, Astrid Bertsche, Micha Loebermann, Hilte Geerdes-Fenge, Robert Fleischmann, Gerd Klinkmann, Patrick Schramm, Sarah Schober, Axel Petzold, Robert Perneczky, Thomas Saller

**Affiliations:** 1 Department of Anesthesiology, Intensive Care Medicine and Pain Therapy, Rostock University Medical Center, 18057 Rostock, Germany; 2 Department of Anesthesiology and Intensive Care Medicine, Jena University Hospital, 07747 Jena, Germany; 3 Department of Anaesthesiology, LMU Hospital, LMU Munich, 81377 Munich, Germany; 4 Department of Pediatrics, Rostock University Medical Center, 18057 Rostock, Germany; 5 Department of Pediatrics, Division of Neuropediatrics, Greifswald University Medical Center, 17475 Greifswald, Germany; 6 Department of Tropical Medicine, Infectious Diseases and Nephrology, Rostock University Medical Center, 18057 Rostock, Germany; 7 Department of Neurology, Greifswald University Medical Center, 17475 Greifswald, Germany; 8 Department of Neurology, Justus Liebig University, 35385 Giessen, Germany; 9 Department I-General Pediatrics, Hematology and Oncology, University Children’s Hospital, Tübingen, Hoppe-Seyler-Str. 1, 72076 Tübingen, Germany; 10 Department of Molecular Neuroscience, The National Hospital for Neurology and Neurosurgery, Queen Square Institute of Neurology, Moorfields Eye Hospital, UCL, London, UK; 11 Departments of Neurology, Ophthalmology and Expertise-Center for Neuro-ophthalmology, Amsterdam UMC, Amsterdam, The Netherlands; 12 Department of Psychiatry and Psychotherapy, LMU Hospital, LMU Munich, 80336 Munich, Germany; 13 German Center for Neurodegenerative Diseases Munich (DZNE), 81377 Munich, Germany; 14 Munich Cluster for Systems Neurology (SyNergy), 81377 Munich, Germany; 15 Sheffield Institute for Translational Neuroscience (SiTrAN), University of Sheffield, Sheffield, S10 2HQ UK; 16 Ageing Epidemiology (AGE) Research Unit, School of Public Health, Imperial College London, London, W6 8RP UK

**Keywords:** COVID-19 (MeSH unique ID D000086382), Biomarkers (D015415), Neurocognitive disorders (D019965), Delirium (D003693), Critical care (D003422)

## Abstract

**Background:**

Compared to intensive care unit patients with SARS-CoV-2 negative acute respiratory tract infections, patients with SARS-CoV-2 are supposed to develop more frequently and more severely neurologic sequelae. Delirium and subsequent neurocognitive deficits (NCD) have implications for patients’ morbidity and mortality. However, the extent of brain injury during acute COVID-19 and subsequent NCD still remain largely unexplored. Body-fluid biomarkers may offer valuable insights into the quantification of acute delirium, brain injury and may help to predict subsequent NCD following COVID-19.

**Methods:**

In a multicenter, observational case-control study, conducted across four German University Hospitals, hospitalized adult and pediatric patients with an acute COVID-19 and SARS-CoV-2 negative controls presenting with acute respiratory tract infections were included. Study procedures comprised the assessment of pre-existing neurocognitive function, daily screening for delirium, neurological examination and blood sampling. Fourteen biomarkers indicative of neuroaxonal, glial, neurovascular injury and inflammation were analyzed. Neurocognitive functions were re-evaluated after three months.

**Results:**

We enrolled 118 participants (90 adults, 28 children). The incidence of delirium [85 out of 90 patients (94.4%) were assessable for delirium) was comparable between patients with COVID-19 [16 out of 61 patients (26.2%)] and SARS-CoV-2 negative controls [8 out of 24 patients (33.3%); *p* > 0.05] across adults and children. No differences in outcomes as measured by the modified Rankin Scale, the Short-Blessed Test, the Informant Questionnaire on Cognitive Decline in the Elderly, and the pediatrics cerebral performance category scale were observed after three months. Levels of body-fluid biomarkers were generally elevated in both adult and pediatric cohorts, without significant differences between SARS-CoV-2 negative controls and COVID-19. In COVID-19 patients experiencing delirium, levels of GFAP and MMP-9 were significantly higher compared to those without delirium.

**Conclusions:**

Delirium and subsequent NCD are not more frequent in COVID-19 as compared to SARS-CoV-2 negative patients with acute respiratory tract infections. Consistently, biomarker levels of brain injury indicated no differences between COVID-19 cases and SARS-CoV-2 negative controls. Our data suggest that delirium in COVID-19 does not distinctly trigger substantial and persistent subsequent NCD compared to patients with other acute respiratory tract infections.

**Trial registration:**

ClinicalTrials.gov: NCT04359914; date of registration 24-APR 2020.

**Supplementary Information:**

The online version contains supplementary material available at 10.1007/s15010-024-02406-7.

## Introduction

Neurocognitive impairment like delirium and neurocognitive deficits (NCD) has been frequently observed after infections with severe acute respiratory syndrome coronavirus 2 (SARS-CoV-2), increasing morbidity and mortality [1–3]. Evidence from post-mortem brain histology and brain imaging point towards neuroinflammation with neuronal, glial and neurovascular injury during COVID-19, contributing to deteriorations of neurocognitive function [4–7]. Primarily, NCD is observed in adults but may also occur in pediatric patients with higher disease severity [8, 9]. However, the extent of a neuroaxonal, glial and neurovascular injury in critically ill patients with COVID-19 and its impact on long-term outcome remains elusive. Body-fluid biomarkers were validated for a variety of brain disorders, including delirium [10–14] and sepsis-associated encephalopathy [15, 16]. Blood biomarkers offer a less invasive alternative to cerebrospinal fluid examination and have potential for routine use [17, 18]. In the context of COVID-19, these biomarkers have been linked to disease severity, morbidity, and mortality [19]. However, their diagnostic and predictive capabilities in COVID-19 for neurocognitive impairment remain unclear.

In a prospective, observational multicenter study, we evaluated the diagnostic value of blood biomarkers for the assessment of neurocognitive impairment among hospitalized adult and pediatric patients with COVID-19 compared to SARS-CoV-2 negative acute respiratory tract infections.

## Methods

### Study design, ethical approval, and trial registration

Shortly after the onset of the COVID-19 pandemic in spring 2020, we initiated a multicenter, prospective, observational study at four academic hospitals in Germany. The study was approved by the responsible Institutional Ethics Committees and was registered prospectively. The STROBE guidelines apply.

### Inclusion and exclusion criteria

Adult and pediatric patients with suspected acute respiratory tract infections admitted to the hospital were eligible. Inclusion criteria comprised adult or pediatric patients of any age, hospital admission with suspicion of a SARS-CoV-2 infection and a Polymerase Chain Reaction for SARS-CoV-2 (PCR) within 48 h after admission, assigning patients to the COVID-19 (PCR positive) or control (PCR negative) group. Exclusion criteria comprised refusal of study participation by the patient or a legal representative, patient referred from another hospital, confirmation of a SARS-CoV-2 later than 48 h after hospital admission, participation in an interventional study or the presence of an acute central nervous system (CNS) condition (e.g. stroke).

### Study **visits and collection samples and data**

**Fig. 1:**
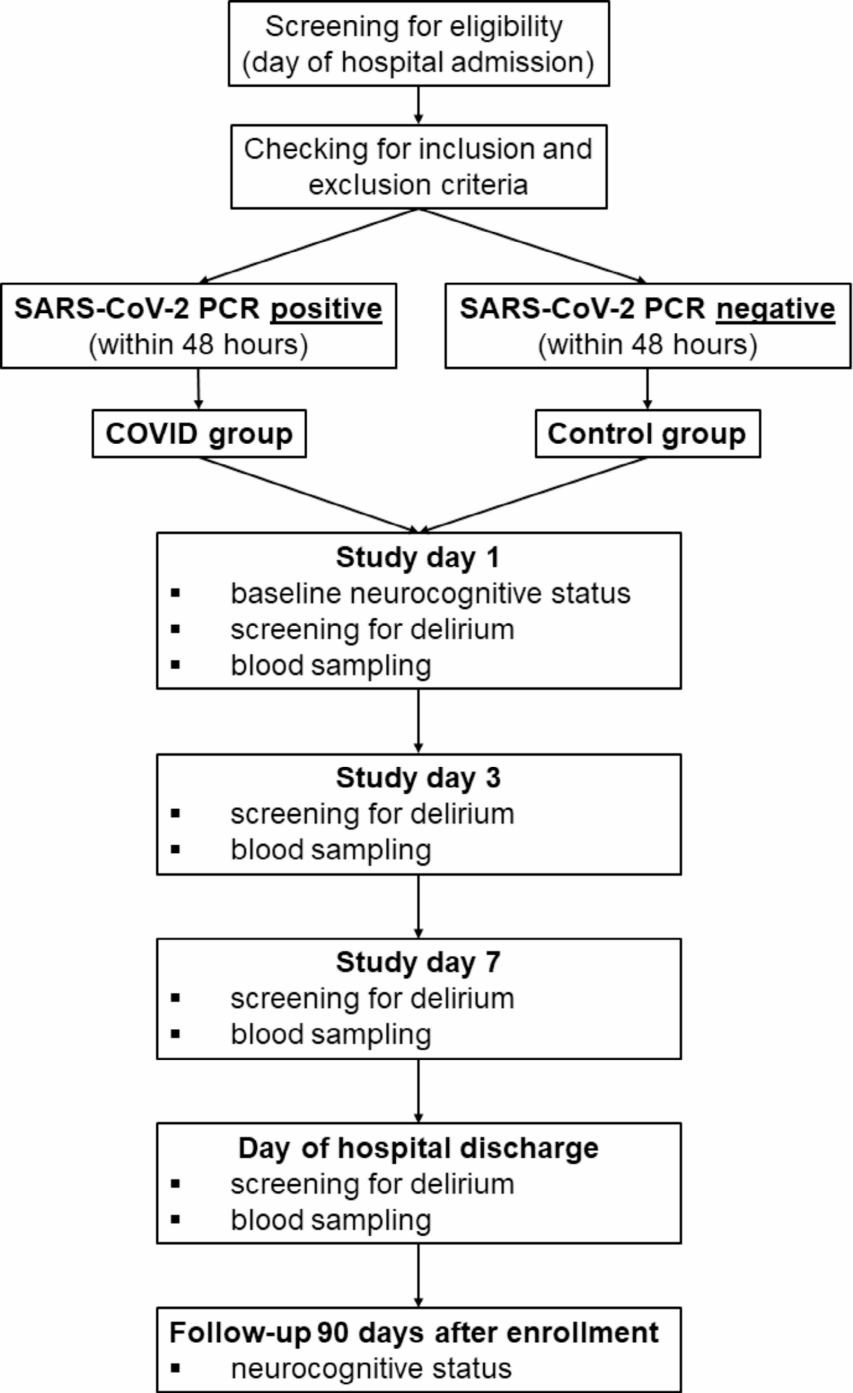
Study flow chart. PCR = Polymerase Chain Reaction

Standardized study visits included clinical, neurological examination and blood sampling at the day of enrollment (day 1), at days 3 and 7 after enrollment and at discharge. Three months after enrollment, standardized telephone interviews were performed (Fig. 1).

Demographic and clinical parameters were collected. At each study visit, the Glasgow Coma Scale (GCS), the Richmond Agitation and Sedation Scale (RASS) and the Sequential Organ failure Assessment (SOFA) scores were assessed. Furthermore, routine laboratory parameters were recorded.

### Assessment of delirium and neurocognitive impairment

Neurocognitive function before the current clinical event and at follow-up was assessed using the Informant Questionnaire on Cognitive Decline in the Elderly (IQCODE), the activities of daily living (ADL) were assessed using the Barthel Index (BI) by the proxy or the patient him- or herself. The Short Blessed Test (SBT), the modified Rankin scale (mRS) and delirium screening was performed by trained clinical assessors. Delirium was detected using the 3D-Confusion Assessment Method (3D-CAM) in non-ICU patients, the Confusion Assessment Method for the Intensive Care Unit (CAM-ICU) and the Intensive Care Delirium Screening Checklist (ICDSC) in ICU patients. In pediatric patients, the pre-school CAM-ICU (psCAM-ICU) was used in children between six months and six years of age. Furthermore, a neuropediatric expert assessed delirium in children below six months. The pediatric CAM-ICU (pCAM-ICU) was used in children five years or older. Three months after study inclusion the neurocognitive status and the ADL of all patients were reevaluated in a standardized telephone interview using the IQCODE, the SBT, the mRS and the BI.

In order to incorporate the relevance of disease severity and neurocognitive impairment, focused sub-group analyses were performed between our patients: first, by comparing patients with COVID-19 and with SARS-CoV-2 negative infections, second, patients with and without delirium independent from SARS-Cov-2 status, third, patients with and without delirium in the COVID cohort and fourths, between patients with and without necessary ICU treatment in the COVID cohort.

### **Body-fluid** biomarkers

Serum and plasma samples were centrifuged (2,000 g, 15 min, 4 °C), aliquoted and stored at -80 °C until analysis. Endothel-Selectin (E-Selectin, R-PLEX Human E-Selectin Assay), Matrix Metalloproteinase-9 (MMP-9, U-PLEX Human MMP-9 Assay), Neurofilament Heavy Chain (NfH, R-PLEX Human Neurofilament H Assay) and Ubiquitine C-terminal Hydrolase-L1 (UCHL-1, Human UCH-L1/PGP9.5 DuoSet kit, R&D systems) were determined with electrochemiluminescence-based immunoassays (ECLIA) according to manufacturer’s recommendations using the MESO Quickplex SQ120 (Meso Scale Discovery (MSD), Rockville, MD, USA). Glial Fibrillary Acidic Protein (GFAP), β-Amyloid 40 and 42, Neurofilament Light Chains (NfL) and Tau protein were determined with the Single-molecule array (Simoa, Neurology 4-Plex, pTau-181) on a HD-1 Analyzer (Quanterix, Lexington, MA, USA). The amino-terminal propeptide of the C-type natriuretic peptide (NT-proCNP, Biomedica Medizinprodukte GmbH, Vienna, Austria) and S100 calcium-binding protein B (S100B, Cloud-Clone Corp., Houston, USA) were measured using sandwich enzyme-linked immunosorbent assays (ELISA). Biomarker samples were measured in duplicates. The analytical error was calculated as coefficient of variation (CV). For samples with an CV > 20% measurements were repeated. Concentrations below the analytical detection limit were replaced with zero values and included in the statistical analysis.

### **Statistical** analyses

Statistical analysis was performed with IBM SPSS Statistics (Version 27, IBM Corp., Armonk, NY, USA). Box plots were created with Sigma Plot 13 (Systat Software, Inpixon, Palo Alto, CA). Normal distribution of continuous data was tested with the Shapiro-Wilk-test and data is given as mean ± standard deviation (SD) or median with interquartile ranges (IQR). For nominal data, absolute and relative frequencies are shown. Distribution of categorical variables between groups was assessed with the Chi-squared test or, if suspected probability was < 5%, the Fisher’s Exact test. Differences between groups were tested for significance by means of the Student’s t-test for normally distributed and the Mann-Whitney U test in case of non-normally distributed data. Age was considered as a putative confounder and a general linear model using an analysis of covariance (ANCOVA), with the biomarker levels as the dependent variable, the particular subgroup as dummy variable and age as covariate was computed. Correlations between continuous variables were calculated with Pearson’s or with Spearman’s rank correlation coefficient as appropriate. A correlation coefficient between 0.1 and 0.39 was considered a weak, between 0.4 and 0.69 a moderate, between 0.7 and 0.89 a strong and ≥ 0.9 a strong correlation. Missing data were not imputed. Statistical significance was defined as *p* ≤ 0.05 and all tests were two-sided. Sample size was estimated for 80 adult and 20 pediatric patients.

## Results

Between April 2020 and March 2021, a total of 90 adult (65 with COVID-19) and 28 pediatric patients (16 with COVID-19) were enrolled. Table 1 shows demographic and clinical data. Table 2 provides demographic data for ICU and non-ICU cohorts. In all cohorts, female gender was comparable.

**Table 1:** Patient demographics, neurocognitive status and biomarker levels of inflammation in adult patients

Demographics and disease severity	COVID	Controls	*P*- values	Delirium	No Delirium	*P*- values	COVID with Delirium	COVID without Delirium	*P*-values
N	65	25	*N/A*	24	61	*N/A*	16	45	*N/A*
Age, median (IQR)	67 (57,79)	74 (57,81)	*0.446*	79 (66,82)	66 (53,75)	***< 0.001***	77 (63,82)	66 (54,75)	***0.022***
Female, n (%)	23 (35.4)	10 (40.0)	*0.684*	6 (25.0)	24 (39.3)	*0.213*	2 (12.5)	18 (40)	***0.044***
Body mass index, mean (SD)	28.9 (5.5)	28.7 (7.1)	*0.570*	28.5 (5.5)	29.1 (6.3)	*0.807*	27.4 (2.7)	29.5 (6.2)	*0.21*
ICU admission, n(%)	31 (47.7)	11 (44.0)	*0.753*	14 (58.3)	23 (37.7)	*0.084*	8 (50)	19 (42.2)	*0.591*
APACHE II Score, mean (SD)	17 (6.0)	21 (9.9)	*0.202*	20.9 (9.6)	16.4 (5.6)	*0.071*	17.6 (5.9)	15.6 (5.1)	*0.472*
SOFA Score day 1, median (IQR)	3 (2,5)	3 (2,10.5)	*0.564*	4 (2,7.5)	3 (2,5)	*0.207*	4 (2,5)	3 (2,5)	*0.159*
SOFA Score day 3, median (IQR)	3 (2,5)	3 (0.5,5)	*0.571*	3 (2,5)	3 (1.5,4)	*0.298*	3 (2,5)	3 (2,4)	*0.476*
SOFA Score day 7, median (IQR)	3(1,7.5)	1 (0,2.5)	***0.011***	2 (0.5,5)	2.5 (0,6.5)	*0.957*	4 (2,6)	3 (0,7)	*0.669*
SOFA Score at hospital discharge, median (IQR)	1 (0,3)	0 (0,3)	*0.538*	0.5 (0,2.3)	1 (0,3)	*0.932*	2 (0,3)	1 (0,2.3)	*0.370*
**Neurocognitive status**									
Barthel Index before admission, mean (SD)	96 (13.3)	83 (28.8)	***< 0.001***	88.3 (23.4)	94.5 (16.1)	***0.013***	96.0 (8.9)	95.6 (14.9)	*0.167*
mRS before admission, median (IQR)	0 (0,0)	0 (0,2)	***0.002***	0 (0,2)	0 (0,0)	***0.002***	0 (0,3)	0 (0,0)	*0.131*
IQCODE day 1, mean (SD)	3.14 (0.34)	3.24 (0.55)	*0.416*	3.22 (0.49)	3.15 (0.38)	*0*,*920*	3.2 (0.4)	3.1 (0.3)	*0.797*
**Comorbidities**									
Cardiovascular, n (%)	42 (64.6)	18 (72.0)	*0.506*	18 (75.0)	39 (63.9)	*0.444*	10 (62.5)	30 (66.7)	*0.763*
Cerebrovascular, n (%)	10 (15.4)	8 (32.0)	*0.078*	10 (41.7)	8 (13.1)	***0.007***	5 (31.3)	5 (11.1)	*0.062*
Pulmonary, n (%)	19 (29.2)	9 (36.0)	*0.534*	9 (37.5)	17 (27.9)	*0.438*	6 (31.3)	11 (24.4)	*0.317*
Renal, n (%)	9 (13.8)	8 (32.0)	*0.072*	10 (41.7)	7 (11.5)	***0.005***	6 (37.5)	3 (6.7)	***0.003***
**Biomarkers of inflammation**									
CRP day 1 [mg/l], median (IQR)	64.0 (26,122)	80.2 (43,164)	*0.488*	56.8 (19,94)	83.1 (45,126)	*0.083*	51.3 (20,89)	93.6 (42,124)	*0.115*
CRP day 3 [mg/l], median (IQR)	58.6 (19,115)	34.0 (22,100)	*0.550*	50.0 (11,97)	52.2 (20,107)	*0.545*	47.0 (9,91)	57.7 (20,118)	*0.204*
CRP day 7 [mg/l], median (IQR)	27.7 (12,88)	22.0 (13,85)	*0.667*	62.5 (18,162)	19.0 (9,49)	***0.009***	62.5 (19,169)	21.0 (9,49)	***0.022***
CRP at hospital discharge [mg/l], median (IQR)	9.1 (5,28)	21.0 (9,67)	*0.190*	17.3 (7,48)	11.4 (5,32)	*0.399*	10.5 (7,23)	8.0 (5,29)	*0.710*
PCT day 1 [ng/ml], median (IQR)	0 (0,0.5)	1 (0,1)	***0.012***	0 (0,1)	0 (0,0)	***0.046***	0.2 (0.1,1.3)	0.1 (0.1,0.4)	*0.479*
PCT day 3 [ng/ml], median (IQR)	0 (0,0.5)	0 (0,0)	*0.110*	0 (0,1)	0 (0,0)	*0.337*	0.1 (0.1,0.6)	0.1 (0.1,0.3)	*0.685*
PCT day 7 [ng/ml], median (IQR)	0 (0,0)	0 (0,0)	*0.868*	0 (0,0)	0 (0,0)	*0.051*	0.2 (0.1,0.5)	0.1 (0.1,0.2)	***0.042***
PCT at hospital discharge [ng/ml], median (IQR)	0 (0,0)	0 (0,0)	*0.301*	0 (0,0)	0 (0,0)	*0.526*	0.1 (0.1,1.7)	0.1 (0.1,0.1)	*0.534*
IL-6 day 1 [pg/ml], median (IQR)	32 (19,88)	26 (11,75)	*0.662*	40 (24,91)	29 (16,69)	*0.243*	40 (29,85)	31 (18,89)	*0.438*
IL-6 day 3 [pg/ml], median (IQR)	29 (9,85)	8 (4,43)	*0.069*	55 (31,88)	15 (7,51)	***0.030***	55 (38,83)	16 (8,93)	*0.075*
IL-6 day 7 [pg/ml], median (IQR)	23 (10,88)	9 (5,25)	*0.112*	64 (20,215)	11 (5,28)	***< 0.001***	76 (20,226)	11 (6,38)	***< 0.001***
IL-6 at hospital discharge [pg/ml], median (IQR)	12 (5,21)	9 (4,12)	*0.490*	12 (8,30)	8 (4,17)	*0.154*	11 (7,15)	12 (4,21)	*0.872*

APACHE II = Acute Physiology and Chronic Health Evaluation; CRP = C-Reactive Protein; ICU = Intensive Care Unit; IL-6 = Interleukin-6; IQCODE = Informant Questionnaire on Cognitive Decline in the Elderly; IQR = Interquartile Range; mRS = Modified Rankin Scale; PCT = Procalcitonin; SD = Standard Deviation; SOFA = Sequential Organ Failure Assessment

In total, 14 blood-based biomarkers were analyzed in detail, forming five different scopes: markers of neurodegeneration (β-Amyloid 40/42, Tau-Protein), endothelial activation (NT-proCNP, MMP-9, E-Selectin), glial activation and injury (S100, GFAP), neuroaxonal injury (NfL, NfH, UCHL-1) and inflammation (CRP, PCT, interleukin-6).

Results will be sequentially presented for adult and pediatric patients. First, (A), we investigated the data from adults with respect to the SARS-CoV-2 status [positive (*n* = 65) vs. negative (*n* = 25)] and the presence (*n* = 24) or absence (*n* = 61) of delirium (B). Subsequently, we compared the results of COVID-19 patients (C) with (*n* = 16) and without delirium (*n* = 45). Furthermore, we categorized data whether treatment on an ICU (D) was required (*n* = 42 vs. *n* = 48). Follow-up data and results for pediatric patients are reported separately.

**Table 2:** Demographics, neurocognitive status and biomarker levels of inflammation in adult patient with and without indication for ICU treatment

	ICU	Non-ICU	*P*-
Demographics and disease severity			
N	42	48	*N/A*
Age, median (IQR)	73 (62,80)	65 (48,79)	***0.028***
Female, n (%)	16 (38.1)	17 (35.4)	*0.965*
Body mass index, mean (SD)	29.8 (6.9)	28.0 (4.8)	*0.158*
APACHE II Score, mean (SD)	18.6 (7.6)	12.8 (5.5)	*0.080*
SOFA Score day 1, median (IQR)	5 (3,11)	2 (2,3)	***< 0.001***
SOFA Score day 3, median (IQR)	5 (3,10)	2 (1,3)	***< 0.001***
SOFA Score day 7, median (IQR)	5 (0,10)	2 (0,3)	***0.013***
SOFA Score at hospital discharge, median (IQR)	0 (0,4)	2 (1,3)	*0.086*
**Neurocognitive status**			
Barthel-Index before admission, mean (SD)	87 (24.9)	96 (8.1)	***0.043***
mRS before admission, median (IQR)	0 (0,1)	0 (0,0)	*0.129*
IQCODE day 1, mean (SD)	3.2 (0.4)	3.2 (0.5)	*0.893*
**Comorbidities**			
Cardiovascular, n (%)	28 (66.7)	32 (66.7)	*1.0*
Cerebrovascular, n (%)	11 (26.2)	7 (14.6)	*0.170*
Pulmonary, n (%)	12(28.6)	16 (33.3)	*0.626*
Renal, n (%)	10 (23.8)	7 (14.6)	*0.285*
**Biomarkers of inflammation**			
CRP day 1 [mg/l], median (IQR)	54.5 (27,142)	74.7 (26,120)	*0.977*
CRP day 3 [mg/l], median (IQR)	63.5 (21,109)	50 (17,100)	*0.277*
CRP day 7 [mg/l], median (IQR)	40.0 (16,101)	18.5 (12,57)	*0.072*
CRP at hospital discharge [mg/l], median (IQR)	21.0 (7,48)	8 (4,27)	***0.045***
PCT day 1 [ng/ml], median (IQR)	0 (0,1)	0 (0,0)	***0.012***
PCT day 3 [ng/ml], median (IQR)	0.5 (0,1)	0 (0,0)	***0.008***
PCT day 7 [ng/ml], median (IQR)	0 (0,1)	0 (0,0)	***0.042***
PCT at hospital discharge [ng/ml], median (IQR)	0 (0,4)	0 (0,0)	*0.199*
IL-6 day 1 [pg/ml], median (IQR)	50 (15,195)	30 (17,44)	*0.118*
IL-6 day 3 [pg/ml], median (IQR)	42 (11,95)	17 (7,54)	*0.125*
IL-6 day 7 [pg/ml], median (IQR)	55 (15,177)	11 (6,29)	***0.004***
IL-6 at hospital discharge [pg/ml], median (IQR)	22 (8,142)	8 (3,12)	***< 0.001***

APACHE II = Acute Physiology and Chronic Health Evaluation; CRP = C-Reactive Protein; ICU = Intensive Care Unit; IL-6 = Interleukin-6; IQCODE = Informant Questionnaire on Cognitive Decline in the Elderly; IQR = Interquartile Range; mRS = Modified Rankin Scale; PCT = Procalcitonin; SD = Standard Deviation; SOFA = Sequential Organ Failure Assessment.

### **Results** for **adult patients with and without Covid-19**

At enrollment, patients with COVID-19 and controls were comparable for age, sex, neurocognitive status (IQCODE at day 1) and cardiovascular, cerebrovascular, pulmonary, and renal comorbidities (each *p* > 0.05, Table 1). According to their APACHE II and SOFA scores, patients were equally distributed within the ICU and normal ward subgroups. However, COVID-19 patients had a significantly higher BI before hospital admission (96 ± 13.3 vs. 83 ± 28.8, *p* < 0.001) and a lower baseline mRS compared to controls [0 (0,0) vs. 0 (0,2), *p* = 0.002].

**Fig. 2:**
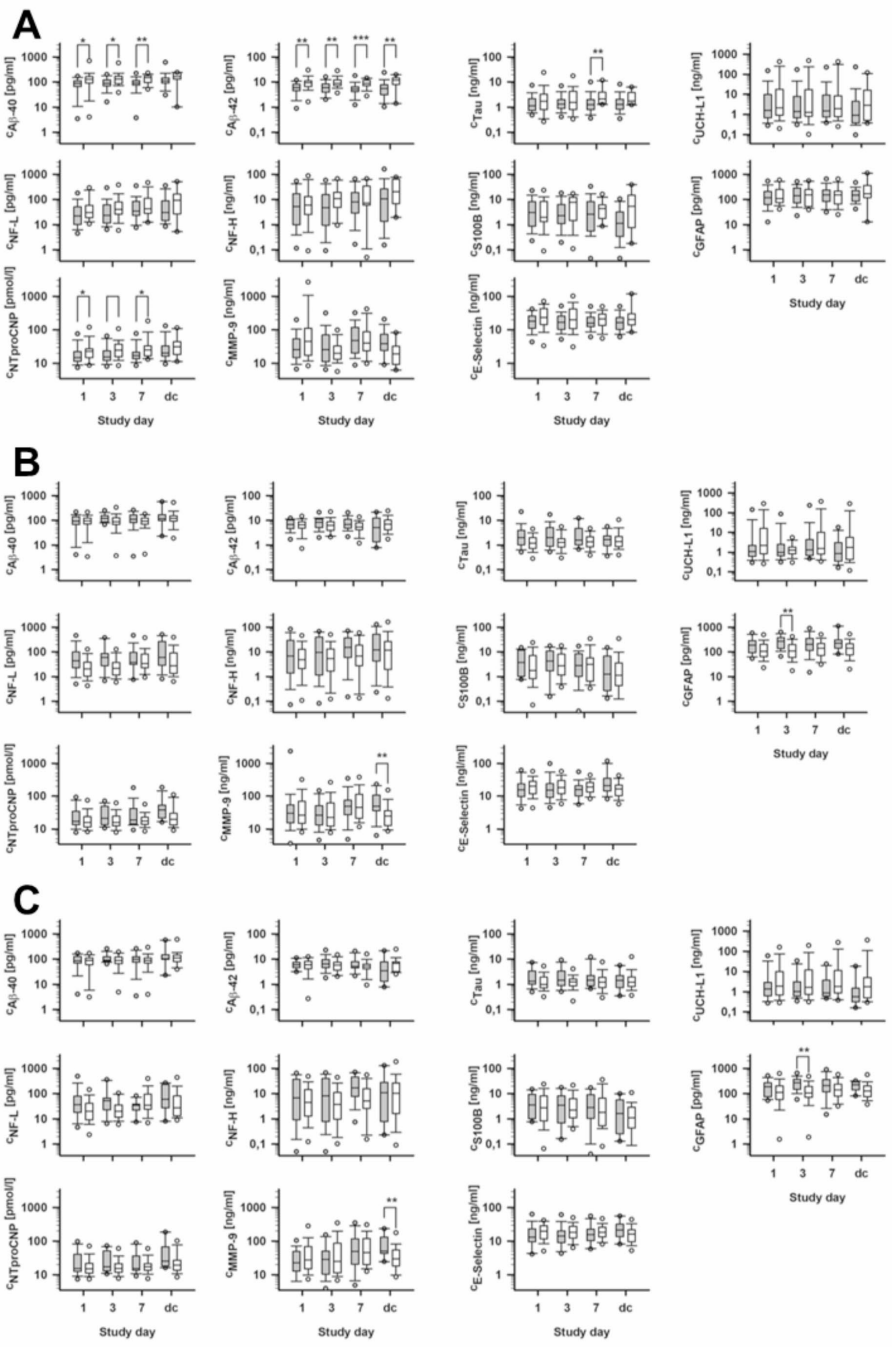
Body-fluid biomarker levels in adult patients at study day 1, 3, 7 and at the time of hospital discharge. (**A**) Comparison between adult patients with COVID-19 (grey box plot) and controls (white box plot), (**B**) Comparison between patients with (grey box plot) and without delirium (white box plot) independent from their SARS-CoV-2 status, (**C**) Comparison between COVID-19 patients with (grey box plot) and without concomitant delirium (white box plot). Boxes and whiskers represent the quartiles together with the median and the 5th and 95th percentiles while symbols indicate data within 1.5x the interquartile range. dc = discharge. * *p* < 0.05; ** *p* < 0.01; *** *p* < 0.001

We found higher values of β-Amyloid 40 and 42 in control patients at study day 1, 7 and at discharge (Additional file 1). Yet, the β-Amyloid 40/42 ratio was comparable. Tau protein in controls was significantly increased at day 7, whereas NT-proCNP was increased at all time points compared to COVID-19 patients (Fig. 2A). An increase in inflammatory parameters tended to be higher and longer in COVID-19 patients.

### **Biomarker levels in** patients **with and without delirium in the total cohort**

Eighty-five patients were analyzed with respect to their delirium status (Table 1). Patients with delirium were older, had a lower BI and a higher mRS before hospital admission and suffered more pre-existing cerebrovascular and renal comorbidities compared to patients without delirium (Table 1). Irrespective of a COVID-19 infection, serum concentration of MMP-9 and GFAP were significantly higher in delirium (Additional file 2, Fig. 2B). Interleukin-6 was increased in patients with delirium [54.6 (31.0,87.8) pg/ml vs. 14.7 (7.4,50.6) pg/ml, *p* = 0.03 at day 3 and 63.6 (20.2, 214.5) pg/ml vs. 11.0 (5.3, 27.6) pg/ml, *p* < 0.001 at day 7]. Furthermore, CRP at day 7 was significantly higher in patients with delirium compared to patients without delirium [62.5 (18,162) vs. 19.0 (9,49) mg/l, *p* = 0.009].

### Biomarker levels in adult COVID-19 patients with and without concomitant delirium

In patients with COVID-19, those with delirium were older (*p* = 0.022), had more renal comorbidities (*p* = 0.003) than patients without delirium and only 12.5% were female (*p* = 0.044). Impaired renal function was frequently associated with delirium (*p* = 0.006). After adjusting for age, COVID-19 patients with delirium had significantly higher GFAP levels at day 3 [270.5 (148.0,375.0) pg/ml vs. 113.0 (70.9,196.0) pg/ml, *p* = 0.021] and higher MMP-9 levels [50.3 (43.1,13.2) µg/ml vs. 28.9 (17.9,47.9) µg/ml, *p* = 0.003] at hospital discharge (Fig. 2C, Additional file 3). CRP levels at day 7 were significantly higher in patients with delirium [62.5 (19,169) mg/l] compared to COVID-patients without delirium [21.0 (9,49) mg/l, *p* = 0.022].

### **Biomarker levels in** adult **COVID-19 patients with and without indication for ICU treatment**

In ICU patients, IL-6 was increased at day 7 [55.2 (15.0,177.5) vs. 11.0 (6.0,29.2) pg/ml, *p* = 0.004] and at hospital discharge [21.7 (7.8,142.0 vs. 8.3 (3.1,12.4) pg/ml, *p* < 0.001], while CRP levels tended to be higher at day 7, but were significantly higher in ICU patients at the day of discharge compared to non-ICU patients [21.0 (7.0,48.0) vs. 8.0 (4.0,27.0) mg/l, *p* = 0.045) as well as MMP-9 at day 3 [45.2(16.9,105.8) vs. 18.6 (10.4,38.7) µg/ml, *p* = 0.002] and S100B at day 1 [6.9 (1.8,12.8) vs. 1.5 (0.8,3.4) ng/ml, *p* < 0.001] after correcting for age (Additional file 4). In contrast, Tau-Protein at day 7 was lower in the ICU cohort [1.3 (0.8,1.8) vs. 1.6 (1.2,3.5) ng/ml, *p* = 0.034].

### Three months follow-up of neurocognitive outcome

Survival, the mRS and the IQCODE was comparable between patients with and without delirium (Table 3), whereas the BI at follow-up was significantly lower in patients with delirium.

**Table 3:** Neurocognitive outcome and survival in COVID-19 patients and controls after three months

	COVID-19	Controls	*p*- Value	Delirium	No Delirium	*p*- Value	COVID-19 with Delirium	COVID-19 without Delirium	*p*-Value
Delirium, n (%)	16 (24.6)	8 (32.0)	*0.513*	N/A	N/A	*N/A*	N/A	N/A	*N/A*
Barthel Index at three months, mean (SD)	92 (20.7)	89 (24.6)	*0.294*	85.6 (24.7)	93,2 (20.4)	***0.014***	91.8 (14.2)	92.2 (22.8)	*0.167*
SBT at three months, median (IQR)	0 (0,4)	2 (0,5.5)	*0.355*	4 (0,6)	0 (0,4)	*0.08*	4 (0,5)	0 (0,4)	*0.206*
mRS at three months, median (IQR)	0 (0,1)	0.5 (0,3)	*0.107*	1 (0,3)	0 (0,1)	*0.051*	0 (0,3)	0 (0,0.3)	*0.450*
IQCODE at three months, mean (SD)	3.10 (0.21)	3.05 (0.25)	*0.067*	3.18 (0.29)	3.05 (0.18)	*0.056*	3 (3,3.4)	3.1 (0.2)	*0.070*
Survival after three months, n (%)	44 (67.7)	20 (80.0)	*0.249*	17 (70.3)	46 (75.4)	*0.665*	11 (69)	33 (73)	*0.088*

IQCODE = Informant Questionnaire on Cognitive Decline in the Elderly; mRS = Modified Rankin Scale; SBT = Short Blessed Test

A correlation analysis between biomarker levels and parameters of neurocognitive outcome revealed multiple, mostly weak to moderate correlations within the subgroups of patients with and without delirium as well as in COVID-19 patients with and without delirium as presented down below. However, patients admitted to the ICU had more delirium [14 (33.3%) vs. 10 (20.8%), *p* = 0.011], a lower BI (80 ± 31.5 vs. 98 ± 8.1, *p* = 0.002), a higher SBT [4 (1.5,6.5) vs. 0 (0.0,4.0), *p* = 0.002] and a higher mRS [0.5 (0.0,3.0) vs. 0 (0.0,1.0), *p* = 0.019] after three months. Furthermore, fewer ICU patients survived [26 (61.9%) vs. 38 (79.2%), *p* = 0.017] (Additional file 5).

### **Correlation between** blood **biomarker concentrations and outcome parameters**

In patients with COVID-19, moderate correlations between the SBT at three months and E-Selectin at day 1 (*r*=-0.437, *p* = 0.007), S100B at day 3 (*r* = 0.553, *p* = 0.011) and S100B at day 7 (*r* = 0.496, *p* = 0.012) were observed. The mRS at three months correlated with NfH at discharge (*r* = 0.404, *p* = 0.024). Furthermore, we observed significant correlations between the BI at three months and NfL (*r*=-0.521, *p* = 0.022) as well as NfH values (*r*=-0.548, *p* = 0.001) at discharge.

In patients with delirium, we observed strong correlations between the SBT at three months and Tau protein at day 3 (*r* = 0.675, *p* = 0.016), E-Selectin at day 7 (*r*=-0.617, *p* = 0.043) and UCHL-1 at discharge (*r* = 0.936, *p* < 0.001) (Additional file 2).

In COVID-19 patients with delirium we observed a strong correlation between the SBT at three months and Tau protein at day 3 (*r* = 0.801, *p* = 0.017) (Additional file 3).

In the ICU cohort, we observed multiple significant weak to strong correlations between biomarkers and outcome, but without a clear pattern (Additional file 4).

### **Demographic** data, **biomarker levels and neurocognitive outcome in the pediatric cohort**

In total, 28 pediatric patients (COVID-19 *n* = 16; control *n* = 12) were included (Additional file 6). Age, sex, and disease severity were comparable between pediatric patients with COVID-19 and controls. However, more pediatric controls were admitted to the ICU (9 (75.0%) vs. 3 (18.8%), *p* = 0.003). There were no differences in inflammatory biomarkers between pediatric COVID-19 patients and controls. The PCPC and the POPC were similar between pediatric COVID-19 patients and controls both at hospital admission and after three months. Only one child with COVID-19 developed delirium.

Because only small sample volumes were available, not all biomarkers of neuroaxonal injury could be assessed in the pediatric cohort. We observed significantly lower levels of MMP-9 at day 1 in pediatric COVID-19 patients compared to controls (28.14 (16.4,38.6) vs. 100.3 (67.2,882.6) µg/ml, *p* = 0.005) and UCHL-1 [2.2 (0.7,16.3) vs. 35.2 (10.7,54.3) ng/ml, *p* = 0.013). E-Selectin, NfH, NT-proCNP and S100B were comparable. Due to the low incidence of delirium in the pediatric cohort, further comparisons were not performed.

## Discussion

Our study found comparable incidence rates of delirium and NCD among COVID-19 and SARS-CoV-2 negative patients of similar disease severity treated in four German university hospitals. In a sub-cohort of pediatric patients, NCD was nearly absent. The concentrations of neuronal and inflammatory biomarkers were elevated above normal mostly without significant differences between COVID-19 patients and SARS-CoV-2 negative controls. In some biomarkers, we encountered higher levels in SARS-CoV-2 negative controls. Our results suggest respiratory tract infections to induce a comparable extend of brain injury, independent of SARS-CoV-2 as a triggering pathogen. In fact, hypoxemia and systemic inflammatory response act as the main triggers of delirium and neuroinflammation and may have played the crucial role in both cohorts. Subsequently, neither the combination nor a single one of the 14 examined biomarkers aids in the detection or prediction of subsequent NCD in COVID-19 patients Patients with COVID-19 and SARS-CoV-2 negative patients showed comparable biomarker levels, which was in line with the clinical incidence of delirium and NCD in both groups. In order to evaluate the discriminative potential of the different blood-based inflammatory and neuronal biomarkers, we performed further sub-group analyses. Neither the discrimination between patients with and without delirium within the total cohort nor between patients with and without delirium in the COVID-19 group was achieved using our biomarker analyses. The discriminative power of blood-based biomarkers to detect clinical phenotypes of delirium in patients with COVID-19 needs further evaluation in large-scale studies. Although the direct neuroinflammatory nature of COVID-19 related neurologic sequelae is often discussed, our results impressively do not promote such a direct neurotropic effect. In COVID-19 research, body-fluid biomarkers have emerged as a promising attempt to detect and quantify brain injury in COVID-19. Hereby, COVID-19 cohorts were mostly compared to either healthy subjects [20–22], patients with acute non-COVID diseases [23], neurodegenerative diseases [24, 25] or other non-infectious pulmonary diseases [26]. Most studies reported elevations of single brain injury markers in COVID-19 patients compared to their controls, underpinning the frequent observations of neurological complications in these patients. According to Girard et al., one third of patients aged > 65 and about 20% of patients aged < 65 showed moderate to severe neurocognitive impairments after a non-COVID community-acquired pneumonia (CAP) [27]. Other authors question increased brain injury in COVID-19 compared to other respiratory tract infections [28]. Their results are confirmed by Needham, who compared biomarkers in COVID-19 patients to patients with influenza and found similar concentrations of GFAP, NfL and Tau protein [29]. All these findings are in line with our results, which show similar extends of biomarker levels and neurological complications between COVID-19 and non-COVID infections of comparable severity. Any kind of systemic infection or sepsis, regardless of its triggering pathogen, may induce neuroinflammation and injury within the CNS [17].

In the presence of delirium, we found elevation of age-corrected blood-levels of GFAP and MMP-9, specifically in COVID-19 patients. Several authors reported elevated GFAP levels in COVID-19 patients with encephalopathy or neurological symptoms [25, 30]. In contrast, several studies investigating GFAP as a perioperative biomarker of brain injury found no clear association with delirium [11, 12, 31, 32]. For MMP-9, data from animal experiments suggested a potential role of reactive oxygen species promoting MMP-9-induced blood brain barrier injury [33]. However, no association between MMP-9 serum levels and neither postoperative delirium nor NCD has been proven yet [34, 35]. Though speculative, but in accordance with the current pathophysiological understanding of delirium, glia cells might play a specific role in the etiology of COVID-19 related neurologic sequalae [36].

During COVID-19, neurological symptoms appear in younger adults and children, mostly consisting of fatigue, myalgia, smell or taste impairments and headache [37]. However, delirium and encephalopathy seem to be rare in pediatric COVID-19 patients [38]. In this context, neuroaxonal injury on the cellular level seems to be limited in children. Geis et al. found no alterations of NfL in a cohort of 148 children with mild to moderate SARS-CoV-2 infection, even in the presence of neurological symptoms [39]. We found lower levels of MMP-9 and UCHL-1 in our pediatric cohort compared to controls. In contrast, Kumar et al. reported on elevated serum concentrations, but without association to neurological symptoms [40]. These results are in line with our observations, suggesting no major brain injury or subsequent NCD in children, even in the acute phase of COVID-19.

### Strength and limitations

The multicentric design promotes a better generalizability of our data compared to other single-center investigations [20, 41–43]. Furthermore, the broad panel of 14 investigated biomarkers allowed for pattern recognition of neuronal, axonal, glial and neurovascular compartments, as well as to quantify the impact of systemic inflammation. Clinical data and biomarker profiles regarding COVID-19 in pediatric patients are sparse, so the present study provides some more insight into the clinical course and neurochemical alterations in this population. Due to limited availability of blood samples in the pediatric cohort not all blood-based biomarkers could be assessed. Furthermore, due to the pandemic situation we did not perform an a priori power analysis due to the uncertainty of the biomarker levels in COVID patients and controls. Due to the missing relevant differences in the biomarker courses, a post hoc power analysis is not suitable in our understanding. Thus, our study cohort may be underpowered. Furthermore, we did not adjust our correlation analyses for multiple comparisons (biomarkers) in order to investigate the diagnostic potential of single blood-based biomarkers, which increases the risk of bias. Clinical, brain imaging and body-fluid biomarker results from well-powered COVID-19 registries might therefore help to verify the results of the present study.

## Conclusions

Our study reveals a similarity in the occurrence of delirium and subsequent NCD among COVID-19 patients and SARS-CoV-2 negative individuals with comparable respiratory tract infections. Notably, in pediatric COVID-19 disease, delirium emerges as a rare event, with a complete absence of subsequent NCD. Our case-control data suggest that delirium in COVID-19 does not distinctly trigger persistent and clinically significant subsequent NCD over and above what is observed for other respiratory tract infections.

## Electronic supplementary material

Below is the link to the electronic supplementary material.

Supplementary Material 1

Supplementary Material 2

Supplementary Material 3

Supplementary Material 4

Supplementary Material 5

Supplementary Material 6

## Data Availability

No datasets were generated or analysed during the current study.

## Abbreviations

3D-CAM: 3 Dimensional Confusion Assessment Method
ADL: Activities of Daily Living
ANCOVA: Analysis of Covariance
APACHE II: Acute Physiology And Chronic Health Evaluation score II
BI: Barthel Index
CAM-ICU: Confusion Assessment Method for the Intensive Care Unit
CAP: Community-acquired Pneumonia
CRP: C-reactive Protein
CV: Coefficient of Variation
Dc: Discharge
E-Selectin: Endothel-Selectin
ECLIA: Electrochemiluminescence Immunoassay
ELISA: Enzyme-linked Immunosorbent Assays
GCS: Glasgow Coma Scale
GFAP: Glial Fibrillary Acidic Protein
ICDSC: Intensive Care Delirium Screening Checklist
ICU: Intensive Care Unit
IL-6: Interleukin-6
IQCODE: Informant Questionnaire on Cognitive Decline in the Elderly
IQR: Interquartile Range
MMP-9: Matrix Metalloproteinase-9
mRS: modified Rankin Scale
NfL/NfH: Neurofilament Light/Heavy Chain Protein
NT-proCNP: N-terminal pro C-type Natriuretic Peptide
PCR: Polymerase Chain Reaction
PCT: Procalcitonin
psCAM-ICU: pre-school CAM-ICU
RASS: Richmond Agitation and Sedation Scale
S100B: S100 calcium-binding Protein B
SBT: Short Blessed Test
SIMOA: Single-molecule Array Immunoassay
SOFA: Sequential Organ Failure Assessment
UCHL-1: Ubiquitine C-terminal Hydrolase L-1
