# Supplementary material for: No substantial neurocognitive impact of COVID-19 across ages and disease severity: a multicenter biomarker study of SARS-CoV-2 positive and negative adult and pediatric patients with acute respiratory tract infections
Source: Infection. 2024 Oct 1;53(2):593–605. doi: 10.1007/s15010-024-02406-7 (PMC11971204; doi:10.1007/s15010-024-02406-7)
Supplement: Supplementary file 1 — Supplementary Material 1 [file 15010_2024_2406_MOESM1_ESM.docx]

**No substantial neurocognitive impact of COVID-19 across ages and disease severity: A multicenter biomarker study of SARS-CoV-2 positive and negative adult and pediatric patients with acute respiratory tract infections**

*Infection*. Johannes Ehler et al. Department of Anesthesiology and Intensive Care Medicine, Jena University Hospital, 07747 Jena, Germany; [johannes.ehler@med.uni-jena.de](mailto:johannes.ehler@med.uni-jena.de)

**Additional File 1**

**Biomarkers in COVID-19 patients versus Controls**

| **Biomarker** |  | **COVID-19** | | | **Control** | | | **p value** |
| --- | --- | --- | --- | --- | --- | --- | --- | --- |
|  |  | median | 25^th^–75^th^percentile | | median | 25^th^–75^th^percentile | |  |
| **β-Amyloid 40**  **[pg/ml]** | day 1 | 88.20 | 66.05 | 111.50 | 128.00 | 90.50 | 163.00 | **0.014** |
|  | day 3 | 87.55 | 69.70 | 121.00 | 128.00 | 83.80 | 167.75 | **0.016** |
|  | day 7 | 89.90 | 74.90 | 113.00 | 148.00 | 94.48 | 177.25 | **0.008** |
|  | discharge | 110.00 | 91.48 | 146.00 | 165.00 | 129.00 | 236.00 | 0.073 |
| **β-Amyloid 42**  **[pg/ml]** | day 1 | 5.95 | 4.46 | 8.13 | 9.20 | 6.55 | 11.05 | **0.002** |
|  | day 3 | 5.96 | 3.94 | 8.38 | 8.76 | 7.24 | 12.00 | **0.002** |
|  | day 7 | 5.09 | 4.19 | 6.50 | 8.73 | 7.13 | 11.80 | **<0.001** |
|  | discharge | 5.53 | 3.13 | 7.70 | 11.80 | 7.97 | 14.20 | **0.023** |
| **CRP  [mg/l]** | day 1 | 64.0 | 26.0 | 122.0 | 80.2 | 43.0 | 164.0 | 0.488 |
|  | day 3 | 58.6 | 19.0 | 115.0 | 34.0 | 22.0 | 100.0 | 0.550 |
|  | day 7 | 27.7 | 12.0 | 88.0 | 22.0 | 13.0 | 85.0 | 0.667 |
|  | discharge | 9.1 | 5.0 | 28.0 | 21.0 | 9.0 | 67.0 | 0.190 |
| **D-Dimer [mg/l]** | day 1 | 1.10 | 0.63 | 2.00 | 2.20 | 0.77 | 4.20 | 0.113 |
|  | day 3 | 0.84 | 0.65 | 1.40 | 1.60 | 1.05 | 4.10 | **0.040** |
|  | day 7 | 0.90 | 0.65 | 1.60 | 1.50 | 1.30 | 4.00 | 0.052 |
|  | discharge | 0.65 | 0.52 | 0.85 | 2.60 | 1.08 | 5.40 | **<0.001** |
| **Endothel-Selectin [ng/ml]** | day 1 | 18.09 | 11.19 | 26.06 | 23.26 | 14.51 | 43.07 | 0.124 |
|  | day 3 | 16.78 | 10.31 | 25.64 | 20.07 | 10.89 | 41.69 | 0.191 |
|  | day 7 | 15.98 | 12.43 | 23.99 | 21.26 | 13.48 | 29.88 | 0.379 |
|  | discharge | 16.38 | 10.33 | 23.27 | 20.28 | 14.04 | 30.14 | 0.482 |
| **GFAP [pg/ml]** | day 1 | 118.00 | 62.60 | 209.00 | 109.00 | 74.95 | 251.75 | 0.687 |
|  | day 3 | 139.00 | 72.80 | 285.50 | 151.00 | 52.40 | 248.00 | 0.728 |
|  | day 7 | 152.00 | 82.25 | 238.00 | 151.00 | 66.15 | 212.75 | 0.702 |
|  | discharge | 148.50 | 85.15 | 225.25 | 170.00 | 118.00 | 361.00 | 0.421 |
| **IL-6 [pg/ml]** | day 1 | 32.40 | 18.70 | 88.40 | 26.30 | 11.20 | 74.55 | 0.662 |
|  | day 3 | 29.05 | 9.49 | 85.10 | 8.27 | 4.41 | 43.15 | 0.069 |
|  | day 7 | 22.50 | 9.86 | 88.30 | 8.88 | 5.47 | 24.75 | 0.112 |
|  | discharge | 11.80 | 5.08 | 21.30 | 8.63 | 4.19 | 12.40 | 0.490 |
| **MMP9 [ng/ml]** | day 1 | 25.70 | 14.77 | 53.60 | 45.19 | 17.29 | 111.77 | 0.063 |
|  | day 3 | 25.62 | 11.30 | 70.61 | 20.03 | 13.29 | 32.95 | 0.258 |
|  | day 7 | 48.01 | 20.12 | 119.55 | 40.57 | 23.79 | 87.24 | 0.941 |
|  | discharge | 38.88 | 24.31 | 73.56 | 19.15 | 9.29 | 32.45 | **0.035** |
| **NfH [ng/ml]** | day 1 | 4.57 | 0.75 | 14.49 | 3.32 | 0.05 | 9.78 | 0.817 |
|  | day 3 | 3.34 | 0.56 | 10.80 | 8.05 | 0.24 | 15.49 | 0.986 |
|  | day 7 | 7.24 | 2.08 | 18.70 | 6.44 | 0.67 | 27.49 | 0.699 |
|  | discharge | 9.76 | 0.70 | 25.08 | 7.66 | 0.00 | 52.36 | 0.919 |
| **NfL [pg/ml]** | day 1 | 21.90 | 10.22 | 50.58 | 30.35 | 18.88 | 58.73 | 0.099 |
|  | day 3 | 23.30 | 11.70 | 61.00 | 39.55 | 22.85 | 74.33 | 0.163 |
|  | day 7 | 34.70 | 23.10 | 83.63 | 42.80 | 28.05 | 109.75 | 0.249 |
|  | discharge | 30.65 | 14.75 | 88.55 | 92.10 | 26.90 | 164.00 | 0.261 |
| **NTproCNP [pmol/l]** | day 1 | 14.90 | 11.56 | 22.40 | 22.96 | 14.85 | 27.18 | **0.045** |
|  | day 3 | 15.46 | 12.19 | 23.59 | 24.58 | 15.77 | 37.34 | **0.008** |
|  | day 7 | 16.94 | 13.68 | 21.38 | 25.38 | 16.73 | 33.39 | **0.022** |
|  | discharge | 20.44 | 16.34 | 33.02 | 31.10 | 18.37 | 44.12 | 0.215 |
| **PCT [ng/ml]** | day 1 | 0.15 | 0.10 | 0.39 | 0.50 | 0.17 | 1.02 | **0.012** |
|  | day 3 | 0.14 | 0.08 | 0.32 | 0.25 | 0.11 | 0.50 | 0.110 |
|  | day 7 | 0.11 | 0.07 | 0.24 | 0.09 | 0.08 | 0.34 | 0.868 |
|  | discharge | 0.08 | 0.06 | 0.11 | 0.11 | 0.08 | 0.22 | 0.301 |
| **S100β-Protein [ng/ml]** | day 1 | 3.01 | 0.86 | 8.17 | 1.93 | 1.31 | 8.29 | 0.801 |
|  | day 3 | 2.28 | 1.10 | 6.33 | 7.68 | 1.45 | 12.22 | 0.086 |
|  | day 7 | 2.56 | 0.56 | 8.21 | 4.40 | 1.75 | 6.03 | 0.244 |
|  | discharge | 1.12 | 0.34 | 3.17 | 5.39 | 0.77 | 14.88 | 0.154 |
| **Tau-Protein [ng/ml]** | day 1 | 1.14 | 0.74 | 2.16 | 1.72 | 0.81 | 3.31 | 0.219 |
|  | day 3 | 1.32 | 0.88 | 1.99 | 1.56 | 0.80 | 4.21 | 0.288 |
|  | day 7 | 1.24 | 0.81 | 1.98 | 2.09 | 1.39 | 3.91 | **0.004** |
|  | discharge | 1.28 | 0.84 | 2.06 | 1.77 | 1.28 | 3.78 | 0.071 |
| **UCHL-1 [ng/ml]** | day 1 | 1.58 | 0.68 | 9.79 | 1.62 | 0.77 | 16.88 | 0.272 |
|  | day 3 | 1.41 | 0.68 | 7.88 | 1.25 | 0.62 | 16.58 | 0.106 |
|  | day 7 | 1.47 | 0.74 | 9.38 | 1.55 | 0.76 | 6.97 | 0.344 |
|  | discharge | 0.91 | 0.38 | 4.48 | 2.88 | 0.50 | 16.24 | 0.309 |
| **WBC [10-9/l]** | day 1 | 5.60 | 4.18 | 9.19 | 10.70 | 7.89 | 15.00 | **<0.001** |
|  | day 3 | 7.04 | 4.53 | 9.64 | 9.12 | 7.52 | 12.20 | **0.007** |
|  | day 7 | 8.42 | 5.38 | 11.38 | 9.36 | 7.49 | 10.15 | 0.298 |
|  | discharge | 7.61 | 5.41 | 9.08 | 8.55 | 6.29 | 9.52 | 0.322 |

CRP C-reactive protein; GFAP Glial Fibrillary Acidic Protein; ; IL-6 Interleukin 6; MMP-9 Matrix Metalloproteinase-9; NfH Neurofilament Heavy Chain; NfL Neurofilament Light Chain; NT-proCNP amino-terminal propeptide of the C-type natriuretic peptide; PCT procalcitonin; S100β S100 calcium-binding protein; UCH-L1 Ubiquitine C-terminal Hydrolase-L1; WBC white blood cell count
