# Supplementary material for: No substantial neurocognitive impact of COVID-19 across ages and disease severity: a multicenter biomarker study of SARS-CoV-2 positive and negative adult and pediatric patients with acute respiratory tract infections
Source: Infection. 2024 Oct 1;53(2):593–605. doi: 10.1007/s15010-024-02406-7 (PMC11971204; doi:10.1007/s15010-024-02406-7)
Supplement: Supplementary file 2 — Supplementary Material 2 [file 15010_2024_2406_MOESM2_ESM.docx]

**No substantial neurocognitive impact of COVID-19 across ages and disease severity: A multicenter biomarker study of SARS-CoV-2 positive and negative adult and pediatric patients with acute respiratory tract infections**

*Infection*. Johannes Ehler et al. Department of Anesthesiology and Intensive Care Medicine, Jena University Hospital, 07747 Jena, Germany; [johannes.ehler@med.uni-jena.de](mailto:johannes.ehler@med.uni-jena.de)

**Additional File 2**

**Biomarkers in Patients with or without Delirium**

| **Biomarker** |  | **Delirium** | | | **No delirium** | | | **p value** |
| --- | --- | --- | --- | --- | --- | --- | --- | --- |
|  |  | median | 25^th^–75^th^percentile | | median | 25^th^–75^th^percentile | |  |
| **β-Amyloid 40**  **[pg/ml]** | day 1 | 92.95 | 68.18 | 142.50 | 96.35 | 70.65 | 125.25 | 0.813 |
|  | day 3 | 119.50 | 86.58 | 155.00 | 87.10 | 67.95 | 126.50 | **0.036** |
|  | day 7 | 111.00 | 81.43 | 165.25 | 93.25 | 69.68 | 124.75 | 0.134 |
|  | discharge | 118.00 | 97.95 | 170.00 | 120.00 | 93.35 | 152.50 | 0.845 |
| **β-Amyloid 42**  **[pg/ml]** | day 1 | 6.86 | 4.61 | 10.21 | 6.51 | 4.84 | 8.91 | 0.984 |
|  | day 3 | 8.32 | 5.03 | 11.18 | 6.28 | 3.94 | 8.71 | 0.119 |
|  | day 7 | 6.74 | 4.84 | 11.00 | 5.36 | 4.19 | 7.93 | 0.103 |
|  | discharge | 5.04 | 1.37 | 12.50 | 7.01 | 4.13 | 10.18 | 0.545 |
| **CRP  [mg/l]** | day 1 | 56.8 | 19.0 | 94.0 | 83.1 | 45.0 | 126.0 | 0.083 |
|  | day 3 | 50.0 | 11.0 | 97.0 | 52.2 | 20.0 | 107.0 | 0.545 |
|  | day 7 | 62.5 | 18.0 | 162.0 | 19.0 | 9.0 | 49.0 | **0.009** |
|  | discharge | 17.3 | 7.0 | 48.0 | 11.4 | 5.0 | 32.0 | 0.399 |
| **D-Dimer [mg/l]** | day 1 | 1.25 | 0.68 | 2.53 | 0.95 | 0.56 | 2.00 | 0.343 |
|  | day 3 | 1.30 | 0.77 | 1.60 | 0.89 | 0.66 | 1.60 | 0.410 |
|  | day 7 | 1.70 | 1.25 | 6.10 | 0.82 | 0.65 | 1.33 | **0.003** |
|  | discharge | 1.20 | 0.63 | 6.40 | 0.70 | 0.55 | 1.00 | 0.114 |
| **Endothel-Selectin [ng/ml]** | day 1 | 15.52 | 9.73 | 23.72 | 20.01 | 11.84 | 28.48 | 0.214 |
|  | day 3 | 15.28 | 9.19 | 24.06 | 18.17 | 11.84 | 30.13 | 0.300 |
|  | day 7 | 15.81 | 10.22 | 20.72 | 18.83 | 13.35 | 26.25 | 0.162 |
|  | discharge | 20.94 | 14.22 | 35.34 | 16.38 | 10.33 | 21.52 | 0.206 |
| **GFAP [pg/ml]** | day 1 | 188.50 | 93.05 | 285.25 | 102.00 | 62.60 | 202.00 | **0.048** |
|  | day 3 | 270.50 | 139.50 | 383.50 | 107.50 | 60.00 | 199.75 | **<0.001** |
|  | day 7 | 203.50 | 113.75 | 356.00 | 141.50 | 71.53 | 222.50 | 0.064 |
|  | discharge | 227.00 | 142.00 | 306.00 | 140.00 | 75.70 | 215.00 | 0.073 |
| **IL-6 [pg/ml]** | day 1 | 40.35 | 24.33 | 91.00 | 29.25 | 16.45 | 69.45 | 0.243 |
|  | day 3 | 54.60 | 30.95 | 87.80 | 14.70 | 7.38 | 50.60 | **0.030** |
|  | day 7 | 63.60 | 20.20 | 214.50 | 11.00 | 5.30 | 27.58 | **<0.001** |
|  | discharge | 12.40 | 8.10 | 29.50 | 8.37 | 4.04 | 16.58 | 0.154 |
| **MMP9 [ng/ml]** | day 1 | 29.97 | 15.55 | 54.31 | 26.30 | 14.94 | 69.24 | 0.769 |
|  | day 3 | 26.39 | 13.40 | 51.57 | 22.38 | 12.24 | 61.55 | 0.894 |
|  | day 7 | 48.70 | 27.32 | 75.40 | 45.18 | 21.40 | 114.04 | 0.931 |
|  | discharge | 48.94 | 35.86 | 108.37 | 24.73 | 12.83 | 36.28 | **0.009** |
| **NfH [ng/ml]** | day 1 | 5.60 | 0.93 | 23.76 | 3.79 | 0.50 | 9.95 | 0.662 |
|  | day 3 | 9.30 | 0.9 | 39.34 | 3.34 | 0.34 | 10.96 | 0.185 |
|  | day 7 | 15.36 | 5.96 | 37.21 | 5.44 | 1.52 | 16.31 | 0.215 |
|  | discharge | 11.50 | 0.84 | 51.04 | 6.67 | 0.35 | 21.87 | 0.712 |
| **NfL [pg/ml]** | day 1 | 43.35 | 22.83 | 99.59 | 20.75 | 10.48 | 38.43 | **0.005** |
|  | day 3 | 59.65 | 27.63 | 86.03 | 21.70 | 11.70 | 37.78 | **0.002** |
|  | day 7 | 37.60 | 30.80 | 94.65 | 34.05 | 22.80 | 81.78 | 0.438 |
|  | discharge | 60.60 | 28.64 | 265.00 | 27.30 | 13.40 | 90.70 | 0.103 |
| **NTproCNP [pmol/l]** | day 1 | 17.20 | 13.57 | 34.53 | 15.98 | 11.66 | 23.77 | 0.163 |
|  | day 3 | 21.07 | 13.55 | 49.19 | 15.84 | 12.42 | 24.71 | 0.073 |
|  | day 7 | 18.82 | 14.41 | 42.03 | 17.37 | 13.69 | 22.83 | 0.176 |
|  | discharge | 38.26 | 21.59 | 53.61 | 19.48 | 13.21 | 30.10 | **0.022** |
| **PCT [ng/ml]** | day 1 | 0.30 | 0.12 | 0.87 | 0.15 | 0.10 | 0.39 | **0.046** |
|  | day 3 | 0.17 | 0.11 | 0.62 | 0.14 | 0.08 | 0.31 | 0.337 |
|  | day 7 | 0.19 | 0.09 | 0.45 | 0.10 | 0.05 | 0.19 | 0.051 |
|  | discharge | 0.06 | 0.05 | 0.17 | 0.09 | 0.06 | 0.16 | 0.526 |
| **S100β-Protein [ng/ml]** | day 1 | 3.69 | 1.03 | 11.59 | 1.77 | 0.83 | 7.13 | 0.221 |
|  | day 3 | 4.22 | 1.73 | 10.95 | 2.70 | 1.15 | 8.07 | 0.485 |
|  | day 7 | 2.81 | 1.03 | 8.43 | 3.12 | 0.65 | 5.66 | 0.853 |
|  | discharge | 1.24 | 0.27 | 6.85 | 1.14 | 0.43 | 3.58 | 0.943 |
| **Tau-Protein [ng/ml]** | day 1 | 2.02 | 1.03 | 3.92 | 1.18 | 0.73 | 1.98 | **0.021** |
|  | day 3 | 2.01 | 0.90 | 5.12 | 1.27 | 0.83 | 1.81 | 0.064 |
|  | day 7 | 1.59 | 1.02 | 4.70 | 1.35 | 0.85 | 2.14 | 0.148 |
|  | discharge | 1.66 | 0.95 | 2.35 | 1.41 | 0.87 | 2.24 | 0.738 |
| **UCHL-1 [ng/ml]** | day 1 | 1.07 | 0.61 | 2.21 | 2.14 | 0.82 | 15.97 | 0.792 |
|  | day 3 | 0.99 | 0.66 | 2.48 | 1.69 | 0.63 | 17.15 | 0.680 |
|  | day 7 | 1.31 | 0.68 | 4.42 | 1.51 | 0.76 | 9.83 | 0.829 |
|  | discharge | 0.85 | 0.35 | 3.10 | 1.37 | 0.41 | 5.58 | 0.680 |
| **WBC [10^-9^/l]** | day 1 | 5.46 | 4.42 | 10.28 | 7.94 | 4.72 | 10.85 | 0.183 |
|  | day 3 | 9.08 | 6.00 | 9.78 | 7.44 | 4.69 | 10.20 | 0.611 |
|  | day 7 | 8.67 | 5.45 | 9.65 | 9.29 | 6.26 | 11.60 | 0.333 |
|  | discharge | 8.41 | 7.11 | 9.96 | 7.44 | 5.44 | 9.29 | 0.194 |

CRP C-reactive protein; GFAP Glial Fibrillary Acidic Protein; ; IL-6 Interleukin 6; MMP-9 Matrix Metalloproteinase-9; NfH Neurofilament Heavy Chain; NfL Neurofilament Light Chain; NT-proCNP amino-terminal propeptide of the C-type natriuretic peptide; PCT procalcitonin; S100β S100 calcium-binding protein; UCH-L1 Ubiquitine C-terminal Hydrolase-L1; WBC white blood cell count
