# Supplementary material for: No substantial neurocognitive impact of COVID-19 across ages and disease severity: a multicenter biomarker study of SARS-CoV-2 positive and negative adult and pediatric patients with acute respiratory tract infections
Source: Infection. 2024 Oct 1;53(2):593–605. doi: 10.1007/s15010-024-02406-7 (PMC11971204; doi:10.1007/s15010-024-02406-7)
Supplement: Supplementary file 3 — Supplementary Material 3 [file 15010_2024_2406_MOESM3_ESM.docx]

**No substantial neurocognitive impact of COVID-19 across ages and disease severity: A multicenter biomarker study of SARS-CoV-2 positive and negative adult and pediatric patients with acute respiratory tract infections**

*Infection*. Johannes Ehler et al. Department of Anesthesiology and Intensive Care Medicine, Jena University Hospital, 07747 Jena, Germany; [johannes.ehler@med.uni-jena.de](mailto:johannes.ehler@med.uni-jena.de)

**Additional File 3**

**Biomarkers in COVID-19 Patients with or without delirium**

| **Biomarker** |  | **COVID-19 with delirium** | | | **COVID-19 without delirium** | | | **p value** |
| --- | --- | --- | --- | --- | --- | --- | --- | --- |
|  |  | median | 25^th^–75^th^percentile | | median | 25^th^–75^th^percentile | |  |
| **β-Amyloid 40**  **[pg/ml]** | day 1 | 85.9 | 69.3 | 122.0 | 89.3 | 60.5 | 109.0 | 0.697 |
|  | day 3 | 91.6 | 83.7 | 123.5 | 86.9 | 67.0 | 119.5 | 0.182 |
|  | day 7 | 98.0 | 75.6 | 120.5 | 88.9 | 70.4 | 110.0 | 0.680 |
|  | discharge | 102.0 | 98.0 | 133.5 | 115.0 | 93.4 | 143.5 | 0.698 |
| **β-Amyloid 42**  **[pg/ml]** | day 1 | 6.0 | 4.6 | 7.4 | 5.9 | 4.2 | 8.1 | 0.912 |
|  | day 3 | 6.5 | 4.6 | 9.2 | 5.7 | 3.7 | 7.3 | 0.314 |
|  | day 7 | 5.1 | 4.6 | 7.2 | 5.1 | 4.1 | 6.3 | 0.636 |
|  | discharge | 3.4 | 1.4 | 6.4 | 6.9 | 4.1 | 7.7 | 0.167 |
| **CRP  [mg/l]** | day 1 | 51.3 | 20.0 | 89.0 | 93.6 | 42.0 | 124.0 | 0.115 |
|  | day 3 | 47.0 | 9.0 | 91.0 | 57.7 | 20.0 | 118.0 | 0.204 |
|  | day 7 | 62.5 | 19.0 | 169.0 | 21.0 | 9.0 | 49.0 | **0.022** |
|  | discharge | 10.5 | 7.0 | 23.0 | 8.0 | 5.0 | 29.0 | 0.710 |
| **Endothel-Selectin [ng/ml]** | day 1 | 13.06 | 9.59 | 22.28 | 19.29 | 11.63 | 25.75 | 0.132 |
|  | day 3 | 14.12 | 9.06 | 19.36 | 17.56 | 12.31 | 27.85 | 0.098 |
|  | day 7 | 15.41 | 10.25 | 21.53 | 16.49 | 13.35 | 22.70 | 0.403 |
|  | discharge | 21.14 | 13.99 | 24.89 | 16.38 | 10.33 | 21.48 | 0.337 |
| **GFAP [pg/ml]** | day 1 | 188.0 | 82.9 | 248.0 | 113.0 | 60.0 | 202.0 | 0.252 |
|  | day 3 | 270.5 | 158.0 | 373.0 | 113.0 | 70.9 | 193.0 | **0.002** |
|  | day 7 | 206.5 | 131.0 | 314.0 | 141.5 | 79.9 | 238.0 | 0.257 |
|  | discharge | 227.0 | 148.5 | 266.5 | 132.5 | 75.7 | 204.0 | 0.091 |
| **IL-6 [pg/ml]** | day 1 | 39.7 | 29.8 | 78.0 | 30.9 | 18.1 | 85.7 | 0.438 |
|  | day 3 | 54.6 | 40.3 | 77.4 | 16.2 | 8.2 | 92.6 | 0.075 |
|  | day 7 | 75.7 | 20.4 | 226.0 | 11.4 | 5.9 | 34.2 | **0.001** |
|  | discharge | 11.2 | 7.6 | 14.6 | 11.6 | 4.6 | 21.3 | 0.872 |
| **MMP9 [ng/ml]** | day 1 | 22.42 | 13.41 | 47.53 | 26.26 | 14.85 | 67.63 | 0.413 |
|  | day 3 | 28.66 | 11.40 | 49.69 | 24.69 | 11.90 | 79.09 | 0.651 |
|  | day 7 | 49.02 | 20.63 | 98.02 | 45.35 | 24.65 | 112.20 | 0.944 |
|  | discharge | 50.31 | 47.25 | 117.18 | 28.92 | 18.74 | 43.81 | **0.017** |
| **NfH [ng/ml]** | day 1 | 6.85 | 0.93 | 37.52 | 3.65 | 0.64 | 9.98 | 0.662 |
|  | day 3 | 8.16 | 0.78 | 39.87 | 3.19 | 0.31 | 9.72 | 0.185 |
|  | day 7 | 17.17 | 7.78 | 45.56 | 4.29 | 1.53 | 13.21 | 0.215 |
|  | discharge | 11.03 | 0.79 | 27.97 | 8.49 | 0.46 | 23.42 | 0.712 |
| **NfL [pg/ml]** | day 1 | 36.6 | 18.9 | 70.8 | 19.5 | 8.9 | 35.3 | 0.052 |
|  | day 3 | 52.5 | 23.4 | 66.9 | 19.8 | 11.7 | 33.1 | **0.035** |
|  | day 7 | 34.8 | 25.6 | 38.2 | 34.1 | 23.9 | 93.9 | 0.436 |
|  | discharge | 60.6 | 28.6 | 193.0 | 25.9 | 13.4 | 81.5 | 0.172 |
| **NTproCNP [pmol/l]** | day 1 | 15.4 | 12.8 | 38.8 | 15.2 | 11.5 | 22.1 | 0.442 |
|  | day 3 | 17.4 | 13.2 | 38.6 | 15.5 | 12.2 | 21.4 | 0.227 |
|  | day 7 | 15.2 | 14.1 | 20.8 | 17.1 | 13.7 | 21.3 | 0.851 |
|  | discharge | 25.2 | 19.1 | 48.4 | 18.9 | 13.7 | 27.2 | 0.090 |
| **PCT [ng/ml]** | day 1 | 0.2 | 0.1 | 0.3 | 0.1 | 0.1 | 0.4 | 0.479 |
|  | day 3 | 0.1 | 0.1 | 0.5 | 0.1 | 0.1 | 0.2 | 0.685 |
|  | day 7 | 0.2 | 0.1 | 0.4 | 0.1 | 0.1 | 0.2 | **0.042** |
|  | discharge | 0.1 | 0.1 | 0.1 | 0.1 | 0.1 | 0.1 | 0.534 |
| **S100β-Protein [ng/ml]** | day 1 | 3.5 | 1.0 | 7.6 | 1.9 | 0.8 | 8.0 | 0.588 |
|  | day 3 | 3.5 | 1.0 | 7.5 | 2.3 | 1.3 | 6.4 | 0.973 |
|  | day 7 | 2.8 | 1.2 | 8.7 | 2.3 | 0.6 | 6.6 | 0.834 |
|  | discharge | 1.6 | 0.3 | 3.4 | 1.1 | 0.5 | 2.8 | 0.959 |
| **Tau-Protein [ng/ml]** | day 1 | 1.3 | 1.0 | 3.0 | 1.0 | 0.7 | 1.8 | 0.055 |
|  | day 3 | 1.5 | 0.9 | 3.0 | 1.2 | 0.9 | 1.6 | 0.307 |
|  | day 7 | 1.5 | 0.9 | 2.0 | 1.2 | 0.8 | 1.9 | 0.464 |
|  | discharge | 1.4 | 0.9 | 2.1 | 1.3 | 0.8 | 2.0 | 0.840 |
| **UCHL-1 [ng/ml]** | day 1 | 1.4 | 0.6 | 2.9 | 1.9 | 0.7 | 9.8 | 0.213 |
|  | day 3 | 1.0 | 0.6 | 3.0 | 1.7 | 0.7 | 13.7 | 0.273 |
|  | day 7 | 0.9 | 0.6 | 3.7 | 1.8 | 0.9 | 11.2 | 0.170 |
|  | discharge | 0.6 | 0.6 | 1.5 | 1.8 | 0.4 | 4.7 | 0.213 |
| **WBC [10-9/l]** | day 1 | 4.5 | 3.6 | 5.5 | 6.4 | 4.6 | 9.3 | **0.008** |
|  | day 3 | 6.6 | 4.7 | 9.2 | 7.2 | 4.6 | 9.7 | 0.616 |
|  | day 7 | 8.3 | 5.1 | 9.3 | 8.8 | 6.2 | 11.6 | 0.369 |
|  | discharge | 8.3 | 6.8 | 9.5 | 7.1 | 5.4 | 9.0 | 0.432 |

ICU intensive care unit; CRP C-reactive protein; GFAP Glial Fibrillary Acidic Protein; ; IL-6 Interleukin 6; MMP-9 Matrix Metalloproteinase-9; NfH Neurofilament Heavy Chain; NfL Neurofilament Light Chain; NT-proCNP amino-terminal propeptide of the C-type natriuretic peptide; PCT procalcitonin; S100β S100 calcium-binding protein; UCH-L1 Ubiquitine C-terminal Hydrolase-L1; WBC white blood cell count
