# Supplementary material for: No substantial neurocognitive impact of COVID-19 across ages and disease severity: a multicenter biomarker study of SARS-CoV-2 positive and negative adult and pediatric patients with acute respiratory tract infections
Source: Infection. 2024 Oct 1;53(2):593–605. doi: 10.1007/s15010-024-02406-7 (PMC11971204; doi:10.1007/s15010-024-02406-7)
Supplement: Supplementary file 4 — Supplementary Material 4 [file 15010_2024_2406_MOESM4_ESM.docx]

**No substantial neurocognitive impact of COVID-19 across ages and disease severity: A multicenter biomarker study of SARS-CoV-2 positive and negative adult and pediatric patients with acute respiratory tract infections**

*Infection*. Johannes Ehler et al. Department of Anesthesiology and Intensive Care Medicine. Jena University Hospital. 07747 Jena. Germany; [johannes.ehler@med.uni-jena.de](mailto:johannes.ehler@med.uni-jena.de)

**Additional File 4**

**Biomarkers in ICU Patients patients versus non ICU patients**

| **Biomarker** |  | **ICU** | | | **No ICU** | | | **p value** |
| --- | --- | --- | --- | --- | --- | --- | --- | --- |
|  |  | median | 25^th^–75^th^percentile | | median | 25^th^–75^th^percentile | |  |
| **β-Amyloid 40**  **[pg/ml]** | day 1 | 98.2 | 75.5 | 136.0 | 91.7 | 56.4 | 132.0 | 0.387 |
|  | day 3 | 91.2 | 79.8 | 126.8 | 96.0 | 69.3 | 135.0 | 0.996 |
|  | day 7 | 91.4 | 75.1 | 124.8 | 103.0 | 76.1 | 159.0 | 0.228 |
|  | discharge | 118.0 | 82.9 | 142.0 | 119.5 | 95.2 | 171.3 | 0.645 |
| **β-Amyloid 42**  **[pg/ml]** | day 1 | 6.2 | 4.7 | 8.2 | 7.1 | 4.2 | 9.3 | 0.473 |
|  | day 3 | 6.4 | 4.7 | 8.4 | 6.5 | 3.9 | 10.5 | 0.755 |
|  | day 7 | 5.7 | 4.3 | 7.8 | 5.7 | 4.6 | 9.7 | 0.460 |
|  | discharge | 5.5 | 3.1 | 9.4 | 7.2 | 3.2 | 10.9 | 0.585 |
| **CRP  [mg/l]** | day 1 | 54.5 | 27.0 | 142.0 | 74.7 | 26.0 | 120.0 | 0.977 |
|  | day 3 | 63.5 | 21.0 | 109.0 | 50.0 | 17.0 | 100.0 | 0.277 |
|  | day 7 | 40.0 | 16.0 | 101.0 | 18.5 | 12.0 | 57.0 | 0.072 |
|  | discharge | 21.0 | 7.0 | 48.0 | 8.0 | 4.0 | 27.0 | **0.045** |
| **D-Dimer [mg/l]** | day 1 | 1.7 | 0.7 | 4.0 | 1.1 | 0.7 | 1.7 | 0.170 |
|  | day 3 | 1.5 | 0.6 | 4.8 | 0.9 | 0.7 | 1.5 | 0.221 |
|  | day 7 | 2.8 | 1.2 | 5.8 | 0.8 | 0.7 | 1.4 | **0.002** |
|  | discharge | 5.3 | 1.6 | 750.9 | 0.7 | 0.5 | 1.1 | **0.016** |
| **Endothel-Selectin [ng/ml]** | day 1 | 14.25 | 8.84 | 28.46 | 21.02 | 15.61 | 28.51 | **0.038** |
|  | day 3 | 15.63 | 8.85 | 25.23 | 18.93 | 14.58 | 29.18 | 0.063 |
|  | day 7 | 15.85 | 10.22 | 23.99 | 20.02 | 14.95 | 25.85 | 0.090 |
|  | discharge | 19.73 | 8.17 | 38.87 | 16.22 | 13.35 | 21.49 | 0.975 |
| **GFAP [pg/ml]** | day 1 | 136.0 | 78.3 | 271.8 | 99.1 | 62.6 | 200.0 | 0.124 |
|  | day 3 | 157.0 | 93.4 | 283.3 | 138.0 | 65.3 | 284.0 | 0.524 |
|  | day 7 | 150.0 | 81.8 | 217.0 | 166.5 | 77.6 | 275.8 | 0.722 |
|  | discharge | 170.0 | 134.0 | 224.5 | 129.0 | 70.8 | 253.8 | 0.403 |
| **IL-6 [pg/ml]** | day 1 | 50.3 | 15.3 | 195.0 | 30.4 | 17.2 | 43.7 | 0.118 |
|  | day 3 | 41.5 | 11.2 | 95.3 | 16.9 | 7.1 | 54.2 | 0.125 |
|  | day 7 | 55.2 | 15.0 | 177.5 | 11.0 | 6.0 | 29.2 | **0.004** |
|  | discharge | 21.7 | 7.8 | 142.0 | 8.3 | 3.1 | 12.4 | **<0.001** |
| **MMP9 [ng/ml]** | day 1 | 44.50 | 23.10 | 80.01 | 19.12 | 12.83 | 68.04 | **0.015** |
|  | day 3 | 45.22 | 16.89 | 105.76 | 18.59 | 10.35 | 38.69 | **0.002** |
|  | day 7 | 57.71 | 26.07 | 120.65 | 35.46 | 16.81 | 59.45 | 0.052 |
|  | discharge | 51.95 | 22.07 | 108.23 | 28.39 | 16.69 | 47.66 | 0.116 |
| **NfH [ng/ml]** | day 1 | 4.9 | 0.9 | 14.7 | 3.1 | 0.1 | 9.9 | 0.086 |
|  | day 3 | 5.7 | 0.9 | 15.9 | 3.3 | 0.1 | 10.8 | 0.178 |
|  | day 7 | 6.9 | 2.8 | 18.8 | 6.9 | 0.5 | 27.1 | 0.189 |
|  | discharge | 13.5 | 2.5 | 38.7 | 6.7 | 0.2 | 23.9 | 0.398 |
| **NfL [pg/ml]** | day 1 | 24.4 | 13.8 | 42.0 | 22.6 | 10.4 | 69.5 | 0.920 |
|  | day 3 | 32.7 | 19.8 | 60.4 | 26.3 | 11.7 | 66.9 | 0.581 |
|  | day 7 | 35.0 | 26.8 | 81.3 | 40.3 | 19.3 | 98.4 | 0.619 |
|  | discharge | 51.3 | 27.1 | 82.0 | 30.6 | 13.3 | 139.0 | 0.408 |
| **NTproCNP [pmol/l]** | day 1 | 14.4 | 10.0 | 23.1 | 17.6 | 13.0 | 24.8 | 0.067 |
|  | day 3 | 15.8 | 12.5 | 26.3 | 17.2 | 13.2 | 26.5 | 0.499 |
|  | day 7 | 15.4 | 13.6 | 27.7 | 18.7 | 15.7 | 25.4 | 0.167 |
|  | discharge | 22.7 | 12.9 | 46.8 | 21.1 | 17.7 | 34.5 | 0.723 |
| **PCT [ng/ml]** | day 1 | 0.4 | 0.1 | 1.2 | 0.2 | 0.1 | 0.4 | **0.012** |
|  | day 3 | 0.5 | 0.1 | 0.8 | 0.1 | 0.1 | 0.2 | **0.008** |
|  | day 7 | 0.2 | 0.1 | 0.7 | 0.1 | 0.1 | 0.2 | **0.042** |
|  | discharge | 0.2 | 0.0 | 3.9 | 0.1 | 0.1 | 0.1 | 0.199 |
| **S100β-Protein [ng/ml]** | day 1 | 6.9 | 1.8 | 12.8 | 1.5 | 0.8 | 3.4 | **<0.001** |
|  | day 3 | 3.8 | 1.0 | 12.9 | 3.5 | 1.2 | 6.8 | 0.426 |
|  | day 7 | 3.1 | 1.2 | 10.3 | 2.3 | 0.5 | 4.4 | 0.073 |
|  | discharge | 1.6 | 0.6 | 5.0 | 1.1 | 0.3 | 3.4 | 0.639 |
| **Tau-Protein [ng/ml]** | day 1 | 1.2 | 0.8 | 2.3 | 1.3 | 0.7 | 2.6 | 0.755 |
|  | day 3 | 1.4 | 0.8 | 2.1 | 1.3 | 0.9 | 2.4 | 0.925 |
|  | day 7 | 1.3 | 0.8 | 1.8 | 1.6 | 1.2 | 3.5 | **0.034** |
|  | discharge | 1.3 | 0.8 | 1.7 | 1.5 | 0.9 | 2.9 | 0.180 |
| **UCHL-1 [ng/ml]** | day 1 | 1.5 | 0.7 | 6.6 | 1.7 | 0.7 | 14.0 | 0.099 |
|  | day 3 | 1.2 | 0.9 | 5.0 | 1.6 | 0.5 | 17.2 | 0.093 |
|  | day 7 | 1.9 | 0.9 | 5.7 | 1.2 | 0.5 | 11.2 | **0.033** |
|  | discharge | 1.3 | 0.9 | 4.8 | 0.8 | 0.4 | 4.5 | 0.412 |
| **-WBC [10-9/l]** | day 1 | 10.2 | 5.0 | 12.8 | 5.7 | 4.3 | 9.0 | **0.005** |
|  | day 3 | 9.3 | 7.0 | 11.7 | 6.6 | 4.2 | 9.1 | **0.001** |
|  | day 7 | 9.7 | 7.7 | 12.2 | 7.1 | 5.3 | 9.4 | **0.002** |
|  | discharge | 8.7 | 7.4 | 11.7 | 6.3 | 5.1 | 8.5 | **0.001** |
| **Lactate** | day 1 | 1.4 | 1.1 | 1.9 | 1.6 | 1.3 | 1.9 | 0.102 |
|  | day 3 | 1.5 | 1.1 | 2.0 | 1.7 | 1.3 | 2.1 | 0.181 |
|  | day 7 | 1.2 | 0.9 | 1.7 | 1.9 | 1.3 | 2.5 | **0.002** |
|  | discharge | 1.3 | 1.1 | 2.4 | 1.8 | 1.4 | 2.2 | 0.427 |

ICU intensive care unit; CRP C-reactive protein; GFAP Glial Fibrillary Acidic Protein; ; IL-6 Interleukin 6; MMP-9 Matrix Metalloproteinase-9; NfH Neurofilament Heavy Chain; NfL Neurofilament Light Chain; NT-proCNP amino-terminal propeptide of the C-type natriuretic peptide; PCT procalcitonin; S100β S100 calcium-binding protein; UCH-L1 Ubiquitine C-terminal Hydrolase-L1; WBC white blood cell count
