# Supplementary material for: No substantial neurocognitive impact of COVID-19 across ages and disease severity: a multicenter biomarker study of SARS-CoV-2 positive and negative adult and pediatric patients with acute respiratory tract infections
Source: Infection. 2024 Oct 1;53(2):593–605. doi: 10.1007/s15010-024-02406-7 (PMC11971204; doi:10.1007/s15010-024-02406-7)
Supplement: Supplementary file 5 — Supplementary Material 5 [file 15010_2024_2406_MOESM5_ESM.docx]

**No substantial neurocognitive impact of COVID-19 across ages and disease severity: A multicenter biomarker study of SARS-CoV-2 positive and negative adult and pediatric patients with acute respiratory tract infections**

*Infection*. Johannes Ehler et al. Department of Anesthesiology and Intensive Care Medicine, Jena University Hospital, 07747 Jena, Germany; [johannes.ehler@med.uni-jena.de](mailto:johannes.ehler@med.uni-jena.de)

**Additional File 5**

**Demographics and neurocognitive status in paediatric patients**

| **Pediatric COVID-19**  **versus Controls** | | **Pediatric COVID-19** | **Pediatric Controls** | **p value** |
| --- | --- | --- | --- | --- |
|  |  | median (IQR) | median (IQR) |  |
| **Demographics and disease severity** | | | | |
| Age [months] | | 2.5 (0.1,15.3) | 3.0 (1.0,11.5) | 0.518 |
| Female, n (%) | | 6 (37.5) | 5 (41.7) | 0.565 |
| ICU admission, n (%) | | 3 (18.8) | 9 (75.0) | **0.003** |
| SOFA Score | day 1 | 1 (0,3) | 1 (0,4) | 0.928 |
|  | day 3 | 0 (0,1) | 0 (0,2) | 0.932 |
|  | day 7 | 0 (0,2) | 0 (0,2) | 1.0 |
|  | discharge | 0 (0,0) | 0 (0,1) | 0.455 |
| **Neurocognitive status and outcome** | | | | |
| PCPC | admission | 1 (1,1) | 1 (1,1) | 0.120 |
|  | 3 months | 1 (1,1) | 1 (1,1) | 0.355 |
| POPC | admission | 1 (1,1) | 1 (1,1) | 0.120 |
|  | 3 months | 1 (1,1) | 1 (1,1) | 0.355 |
| Delirium | n (%) | 1 (6.3) | 0 | 0.386 |

ICU Intensive Care Unit; IQCODE Informant Questionnaire on Cognitive Decline in the Elderly; IQR Interquartile Range; mRS Modified Rankin Scale; PCPC Pediatric Cerebral Performance Category Scale; POPC Pediatric Overall Performance Category; SD Standard Deviation; SOFA Sequential Organ Failure Assessment
