# Supplementary material for: No substantial neurocognitive impact of COVID-19 across ages and disease severity: a multicenter biomarker study of SARS-CoV-2 positive and negative adult and pediatric patients with acute respiratory tract infections
Source: Infection. 2024 Oct 1;53(2):593–605. doi: 10.1007/s15010-024-02406-7 (PMC11971204; doi:10.1007/s15010-024-02406-7)
Supplement: Supplementary file 6 — Supplementary Material 6 [file 15010_2024_2406_MOESM6_ESM.docx]

**No substantial neurocognitive impact of COVID-19 across ages and disease severity: A multicenter biomarker study of SARS-CoV-2 positive and negative adult and pediatric patients with acute respiratory tract infections**

*Infection*. Johannes Ehler et al. Department of Anesthesiology and Intensive Care Medicine. Jena University Hospital. 07747 Jena. Germany; [johannes.ehler@med.uni-jena.de](mailto:johannes.ehler@med.uni-jena.de)

**Additional File 6**

**Biomarkers in pediatric COVID-19 patients versus Controls**

| **Biomarker** |  | **COVID-19** | | | **Controls** | | | **p value** |
| --- | --- | --- | --- | --- | --- | --- | --- | --- |
|  |  | median | 25^th^–75^th^percentile | | median | 25^th^–75^th^percentile | |  |
| **CRP  [mg/l]** | day 1 | 16.2 | 8.5 | 76.3 | 47.6 | 14.2 | 109.7 | 0.241 |
|  | day 3 | 18.6 | 7.9 | 64.5 | 12.4 | 6.6 | 78.9 | 0.882 |
|  | day 7 | 8.6 | 4.2 | 10.2 | 5.6 | 1.7 | 59.2 | 1.000 |
|  | discharge | 8.7 | 5.6 | 17.2 | 13.6 | 3.0 | 36.0 | 0.589 |
| **D-Dimer [mg/l]** | day 1 | 1.5 | 0.8 | 2.4 | 1.2 | 0.7 | 1.8 | 0.612 |
|  | day 3 | 1.4 | 0.5 | 3.0 | 0.7 | 0.4 | 1.0 | 0.081 |
|  | day 7 | 1.0 | 0.7 | 2.2 | 0.7 | 0.5 | 1.4 | 0.480 |
|  | discharge | 1.1 | 0.9 | 2.3 | 0.6 | 0.4 | 1.2 | 0.231 |
| **Endothel-Selectin [ng/ml]** | day 1 | 45.70 | 28.84 | 57.50 | 32.62 | 28.42 | 123.69 | 1.0 |
|  | day 3 | 29.90 | 28.06 | 41.97 | 39.32 | 20.79 | 85.17 | 0.923 |
|  | day 7 | 19.93 | 12.23 | 22.34 | 40.28 | 19.41 | 63.82 | 0.157 |
|  | discharge | 18.58 | 10.80 | 26.36 | - | - | - | N/A |
| **IL-6 [pg/ml]** | day 1 | 10.1 | 3.0 | 19.0 | 41.4 | 8.9 | 69.6 | 0.055 |
|  | day 3 | 2.7 | 2.7 | 4.6 | 7.6 | 3.1 | 12.6 | 0.111 |
|  | day 7 | 3.3 | 3.0 | 3.5 | 4.1 | 2.2 | 5.7 | 0.480 |
|  | discharge | 2.7 | 2.7 | 2.7 | 5.5 | 2.4 | 24.2 | 0.241 |
| **MMP9 [ng/ml]** | day 1 | 28.14 | 16.36 | 38.60 | 100.28 | 67.18 | 882.57 | **0.005** |
|  | day 3 | 20.23 | 11.60 | 25.26 | 28.88 | 21.64 | 61.22 | 0.102 |
|  | day 7 | 41.75 | 20.22 | 41.89 | 75.55 | 25.86 | 1062.00 | 0.480 |
|  | discharge | 12.44 | 11.22 | 13.65 | - | - | - | N/A |
| **NfH [ng/ml]** | day 1 | 4.1 | 1.6 | 24.5 | 1.2 | 0.6 | 4.2 | 0.386 |
|  | day 3 | 10.4 | 4.3 | 16.5 | 2.2 | 1.0 | 17.5 | 0.433 |
|  | day 7 | 15.7 | 5.7 | 25.7 | 2.6 | 0.5 | 44.4 | 0.564 |
|  | discharge | 8.0 | 1.7 | 25.3 | - | - | - | N/A |
| **NTproCNP [pmol/l]** | day 1 | 79.7 | 32.0 | 132.1 | 42.1 | 33.1 | 59.9 | 0.606 |
|  | day 3 | 74.3 | 22.5 | 120.9 | 51.8 | 35.6 | 70.9 | 1.0 |
|  | day 7 | 31.8 | 30.5 | 139.5 | 43.0 | 37.1 | 204.1 | 0.297 |
|  | Discharge | - | - | - | - | - | - | N/A |
| **PCT [ng/ml]** | day 1 | 0.2 | 0.1 | 1.4 | 0.4 | 0.1 | 1.6 | 0.620 |
|  | day 3 | 0.8 | 0.1 | 2.3 | 0.3 | 0.1 | 0.4 | 0.546 |
|  | day 7 | 0.1 | 0.1 | 0.6 | 0.1 | 0.1 | 0.3 | 0.221 |
|  | discharge | 0.1 | 0.1 | 0.1 | 0.1 | 0.1 | 0.2 | 0.929 |
| **S100β-Protein [ng/ml]** | day 1 | 6.9 | 3.0 | 15.8 | 11.8 | 4.9 | 13.0 | 0.564 |
|  | day 3 | 7.5 | 3.2 | 20.4 | 9.2 | 3.9 | 13.8 | 0.806 |
|  | day 7 | 5.7 | 0.4 | 7.5 | - | - | - | N/A |
|  | discharge | - | - | - | - | - | - | - |
| **UCHL-1 [ng/ml]** | day 1 | 2.2 | 0.7 | 16.3 | 35.2 | 10.7 | 54.3 | **0.013** |
|  | day 3 | 7.1 | 1.1 | 209.3 | 22.1 | 2.4 | 82.9 | 0.564 |
|  | day 7 | 141.8 | 9.0 | 529.7 | 114.7 | 2.2 | 555.9 | 0.624 |
|  | discharge | 243.1 | 1.8 | 484.4 | - | - | - | N/A |
| **WBC [10-9/l]** | day 1 | 9.2 | 3.8 | 12.6 | 14.1 | 8.7 | 22.4 | **0.014** |
|  | day 3 | 6.4 | 2.3 | 9.5 | 8.7 | 5.7 | 15.2 | 0.056 |
|  | day 7 | 8.0 | 4.2 | 15.4 | 10.2 | 8.4 | 12.0 | 0.809 |
|  | discharge | 3.9 | 2.0 | 7.5 | 8.7 | 8.1 | 15.1 | **0.014** |

CRP C-reactive protein; GFAP Glial Fibrillary Acidic Protein; IL-6 Interleukin 6; MMP-9 Matrix Metalloproteinase-9; NfH Neurofilament Heavy Chain; NT-proCNP amino-terminal propeptide of the C-type natriuretic peptide; PCT procalcitonin; S100β S100 calcium-binding protein; UCH-L1 Ubiquitine C-terminal Hydrolase-L1; WBC white blood cell count
